# Patient Experiences With Obstetric Counseling on Fetal Malpresentation

**DOI:** 10.7759/cureus.52683

**Published:** 2024-01-21

**Authors:** Malini S Ramaiyer, Bethlehem Lulseged, Shannon Glynn, Cybill Esguerra

**Affiliations:** 1 Obstetrics and Gynecology, Johns Hopkins University School of Medicine, Baltimore, USA

**Keywords:** prenatal care, patient counseling, ceserean section, external cephalic version, fetal malpresentation

## Abstract

Introduction

Fetal malpresentation is a complication of pregnancy in which the fetus does not present cephalically as required for vaginal birth. After a diagnosis is made, management options include cesarean section (CS) or external cephalic version (ECV). ECV is a procedure in which providers attempt to manually maneuver the fetus to cephalic position, allowing patients to attempt vaginal birth. Selecting between CS or ECV can be a complex and stressful decision, yet literature exploring patient perspectives on counseling of these options is limited. This study aims to describe patient perspectives on decision-making when diagnosed with fetal malpresentation.

Methods

We included English-speaking pregnant patients greater than 18 years of age diagnosed with malpresentation at 35-37 weeks' gestation. Patients who previously underwent CS or had maternal or fetal contraindications besides malpresentation to vaginal birth requiring CS were excluded. Semi-structured interviews were conducted with participants from four obstetric clinics in Baltimore, Maryland, at time of diagnosis. Themes were derived using data analysis in NVivo 11 (released 2015, Lumivero, USA).

Results

We recruited 10 participants (median age = 32 years, 90% Caucasian, 70% nulliparous, 50% chose ECV). We categorized our findings into the following themes: (1) facilitators and (2) barriers to deciding on malpresentation management, (3) participant priorities and values, and (4) other methods of malpresentation management. The participants identified incorporation of statistics and medical history into counseling as facilitators and the lack of information about ECV as a significant barrier. The participants prioritized fetal safety and, among those who chose ECV, a desire to avoid CS. Chiropractors, acupuncture, and moxibustion were identified as valuable additional methods of malpresentation management.

Conclusion

Overall, patients desire more information about ECV when diagnosed with fetal malpresentation. Uncertainty about ECV safety is a barrier to deciding between management options. Based on our findings, obstetric providers should provide comprehensive counseling on ECV and CS. Counseling should aim to demystify ECV and quantify risk in a patient-specific context. This will allow patients to make an informed decision on the management of fetal malpresentation that aligns with their goals for pregnancy.

## Introduction

Fetal malpresentation affects 3-4% of deliveries worldwide [1]. Over 85% of pregnant individuals diagnosed with fetal malpresentation in the third trimester are delivered by cesarean section (CS) [2]. Currently, the standard management of non-vertex presentation (including breech, transverse, and oblique lie) in the United States (US) is trial of external cephalic version (ECV) or scheduled CS. The rate of vaginal delivery of a malpresenting singleton has decreased as the number of practitioners with the skill to safely perform this procedure has decreased [3]. For most low-risk pregnancies, CS presents more maternal risk compared to vaginal birth [2,4-6]. In the US, 17% of CS are indicated by fetal malpresentation [2].

ECV is a viable option for malpresentation management in which providers attempt to manually maneuver the fetus to cephalic position. If successful, ECV allows individuals diagnosed with malpresentation to attempt vaginal birth [7]. Complications of ECV are rare (<1%) but include placental abruption, rupture of membranes, umbilical cord prolapse, maternal-fetal hemorrhage, and stillbirth [8]. Fetal decelerations are not uncommon during ECV but typically resolve once the procedure is paused or discontinued. Persistent bradycardia may necessitate emergency CS. A 2016 study found that individuals who underwent a successful ECV had decreased rates of adverse obstetric outcomes and healthcare utilization compared to individuals who did not undergo ECV [8]. Despite this, less than half of individuals with fetal malpresentation undergo ECV [7]. It is unclear whether the low rate of ECV utilization stems from patients’ true preference for CS, insufficient provider counseling, or if there are other factors influencing patients’ decisions [9-11]. Previous studies have investigated external factors affecting patients’ decision-making process in selecting ECV versus CS. Through interviews with providers, Rosman et al. identified several determinants of low rates of ECV utilization in the Netherlands [11]: while a vast majority of Dutch professionals consider ECV as a viable intervention to prevent CS and endorse options counseling to increase ECV utilization, only 2/3 of professionals believed that it was their responsibility to provide counseling. Similar research tailored to the obstetric provider and patient population in the US is lacking. Through interviews with pregnant individuals diagnosed with fetal malpresentation, this study aims to describe facilitators and barriers to malpresentation management and options counseling in the US.

## Materials and methods

Recruitment

From March 2022 to September 2022, we recruited individuals greater than 18 years of age receiving prenatal care at four different obstetric clinics within the Johns Hopkins Medicine health system who were diagnosed with fetal malpresentation by ultrasound between 35 and 37 weeks. This study was deemed exempt by the Institutional Review Board due to the low risk of participation and confidentiality of the interviews (approval no. IRB00317079). The participants were excluded if they were non-English speaking, had previously undergone CS, or had maternal or fetal diagnoses aside from malpresentation requiring delivery via CS (i.e., placenta previa). The participants were primarily identified through daily reviews of prenatal visit charts. In addition, obstetric providers at each of the four study sites were made aware of the research study and asked to refer patients who had been diagnosed with fetal malpresentation. Routine prenatal care was provided by OB/GYN physicians and mid-level providers. It is important to note that the providers counseling on malpresentation management during prenatal visits were not necessarily the same providers performing the subsequent ECV or CS due to the nature of group practice. Furthermore, some prenatal care providers work only in the ambulatory setting and do not perform ECVs or CSs. Potential participants were contacted via email and phone and were screened for eligibility criteria before being consented. The consent and interview processes were conducted remotely. The participants were recruited until the interviews reached thematic saturation, a frequently utilized method for estimating sample sizes for qualitative research. Thematic saturation is defined by each subsequent interview yielding little to no new themes as the research team reads through and codes each new transcript. 

Interviews

Interviews were conducted and recorded over Zoom by one team member (MR) with training in qualitative methods. The participants kept their video off, and recordings were stored in a secure location. Afterwards, the same team member transcribed and de-identified the data for analysis. The participants were interviewed after the point of diagnosis of malpresentation before the individuals underwent either ECV or CS. Interviews lasted 30-60 minutes and were semi-structured in nature. The interview guide can be found in Table 1. Questions were open-ended, and the topics covered were counseling received from providers, patients’ understanding of their options for management, facilitators and barriers to choosing between ECV and CS, and expectations for either ECV or CS. 

**Table 1:** Semi-structured interview guide The table presents questions and prompts asked to participants during interviews. Questions were focused on counseling and decision-making regarding fetal malpresentation management.

	Question
1.	Introduction of the researcher and recap the purpose of the study. Inform patients that we will be recording the interview in order to create a transcript of the interview. All information will be kept confidential, stored securely, and de-identified.
2.	Do you have any questions or concerns about the study?
3.	What is your understanding of fetal malpresentation and how it affects your options for delivery?
4.	What is your understanding of the management of fetal malpresentation?
5.	What do you know about these different options?
6.	ECV can be associated with rare complications. Which of the following adverse events, if any, were most impactful in your decision-making process for the management of fetal malpresentation: pain control, premature rupture of membranes, placental abruption, umbilical cord prolapse, persistent fetal bradycardia requiring emergency C-section, maternal-fetal hemorrhage, or stillbirth?
7.	What information was most helpful in making your choice?
8.	When making decision concerning obstetric care, how do you prefer information to be presented? When receiving information, do you prefer: numbers, words, or pictures? Do you think a mixture is helpful?
9.	Are there any information or resources you wish you received making your decision?
10.	What other sources are you using to make your decision? (Ex. Family, friends, doula, internet, books) preparation upcoming?
11.	Have you made a decision regarding management of fetal malpresentation?
12.	(If proceeding with ECV) What are your expectations for the ECV?
13.	Do you have any specific concerns about the procedure?

Analysis

Interview transcripts were uploaded to NVivo 11 (released 2015, Lumivero, USA) for qualitative thematic analysis. Three members of the research team (MR, BL, and SG) adopted an inductive approach to content analysis, individually reading the transcripts and developing an initial framework. The research team reviewed and refined this initial coding structure over several meetings, before identifying subthemes that fell under these broader themes and then applied the codes to transcripts. Recruitment occurred concurrently with analysis, and with each new interview transcript, new themes were added to the coding framework. Recruitment stopped as the analysis of new interviews no longer yielded new themes, defined as thematic saturation.

Subsequently, all three researchers coded all interview transcripts and met throughout the analysis process to compare coding and ensure consistency. Disagreements in analysis were resolved through discussion and adjustment of themes and their definitions. Consensus was obtained on a final coding for each of the transcripts with 100% agreement across coders, and the main themes were identified from the frequency of codes applied to the transcripts.

## Results

A total of 52 participants were approached based on the chart review and provider referral to participate via phone call. Among these 52 individuals, 24 participants were successfully contacted, from which 10 agreed to participate and completed an interview. Among the 14 participants who were approached and did not participate, four participants’ fetuses spontaneously converted to vertex position and 10 declined to participate due to lack of time or availability. Participant demographics can be found in Table 2. Five participants opted for ECV while five opted for CS. The median age was 32 years (range: 26-35 years). Nine of the participants identified as Caucasian, and one identified as Black/African American. Seven of the participants were primiparo.

**Table 2:** Demographics of individuals diagnosed with fetal malpresentation in the study The data has been represented as N, %. ECV: external cephalic version, CS: cesarean section

Participant demographics (n = 10)	
Choice of malpresentation management	
ECV	5, 50%
CS	5, 50%
Race	
White	9, 90%
Black	1, 10%
Ethnicity	
Hispanic or Latino	0, 0%
Not Hispanic or Latino	10, 100%
Age	
18-24	0, 0%
25-34	8, 80%
35-49	2, 20%
Primiparous?	
Yes	7, 70%
No	3, 30%

The most prominent themes were (1) facilitators' decision on malpresentation management, (2) barriers to decision on malpresentation management, (3) priorities and values for participants, and (4) methods of malpresentation management beyond ECV or CS. Subthemes identified from the participant interviews are described below. Themes, subthemes, and representative quotes from interviews can be found in Table 3.

**Table 3:** Identified themes and participant quotes

Theme		Quotes
1.	Facilitators' decision on fetal malpresentation management	
1a.	Subtheme - Desire for statistics related to procedure success rates and risk of complications during counseling	“My husband and I are pretty risk averse, which is why we decided to do the... We did early genetic testing. As opposed to waiting and doing like the typical blood tests later in the pregnancy, we did the cell free DNA. Uhm, so you know that 50% success rate, coupled with the increased risk that I have, given that the placenta is anterior. With the additional necessity of having an epidural anyways, I just didn't think that [CS] was the best option for us.”
1b.	Subtheme - Incorporation of medical and pregnancy history into decision making	“We did like 7 years of IVF to get to this point, but it's like I'm just not sure I want to like throw in an added complication. And like hearing that it's an epidural too, I don't know. I just feel like I'm leaning more towards not doing it, then I am doing it.”
		“[When] my son, my first baby came, he was just preterm at 36 weeks and six days, and so I'm worried about going into [this] labor early before a scheduled C-section. So I wanted to see if I could get her to turn because that would make things easier. Maybe prevent the C-section, but also make it safer for me generally if I was going to have early labor.”
2.	Barriers to decision on fetal malpresentation management	
2a.	Subtheme - Lack of information on ECV	“This is newer to me… I didn't have the time to know what questions to ask in the appointment, so I didn't get all the answers I needed in that appointment, and some of it does go into the actual percentage of… what can turn into like an emergency C-section and what percent chance would it send me into like early labor? Who can even be in the room? I didn't even know my husband could be in the room.”
		“It’s basically you have the option of doing ECV. So I did the research, figured out what it was. I mean, I was told somebody is going to lift the baby butt out of your pelvis, and then there's going to be another provider who is turning baby's head, and they're going to basically just try to push. But there was information that I felt like should have been maybe standard to be told that I wasn't told until I asked about it.”
		“Yeah it was mostly do you have questions as opposed to alright so this is what will happen... I feel like especially if this is your first baby and if you've just had a growth and you just found out to say that you're breech and now you're meeting with the doctor on Thursday and you haven't had time to wrap your head around it. And they're like, alright, what questions do you have? And you're like I don't know what questions I'm supposed to have.”
3.	Priorities and values of participants	
3a.	Subtheme - Maternal prioritization of fetal safety	“My only, I'm just like, you know, a little worried about trying to do an ECV. It feels a little counterintuitive because if the baby is in this position, I'm wondering if there's a reason behind it, and then we're trying to do something against it. Uhm, that's kind of more of a feeling you know?”
		“I guess my one concern and I don't have any actually evidence to back this up, but my concern is that maybe she can't turn because the cord is wrapped around her neck and I’m wondering whether they can check for that, and if that's the case, if that causes extra complications.”
	Subtheme - Desire to avoid C-section	“I am worried about the recovery from that because, especially because I have a toddler… And I think it just feels a little more scary to go under surgery, to me, since I've already had one vaginal birth and I know what that was like.”
4.	Methods of malpresentation management beyond ECV and CS	
		“Yeah, I've received chiropractic care at various points in my life and it's made a huge difference in terms of like symptomatic relief and not just treating the symptoms. If I had seen my regular provider, they would have done everything they knew, in their power to do, which would have been symptom management. But I found that chiropractic care can sometimes get to the source quicker and provide relief without masking the root cause.”
		“I don't know if it was just for stress relief, but they also did actual acupuncture. By putting needles in me, I'd never experienced acupuncture before, so that was interesting. He did not flip, but that's not necessarily meaning that the acupuncture doesn't work, it just means it didn't work for him.”
		“Another alternative method that I attempted was the flipping babies or spinning babies or something. I went onto YouTube and looked at videos and my husband and I got a birthing ball and we were on the floor trying to do all these things.”

Theme 1: facilitators' decision on malpresentation management

Subtheme: Desire for Statistics Related to Procedure Success Rates and Risk of Complications During Counseling

The most common subtheme among all participants was expressed appreciation for quantitative data during counseling by providers. The participants described that their decision-making was facilitated by discussion of success and complication rates. For participants that opted for CS, knowing the risks and benefits of surgery in the context of their specific medical history allowed them to choose CS with more certainty. Specifically, participants pointed to the 50% chance of success for ECV, quoted by providers, as a factor in their decision (Table 2).

“For me, the stats are really helpful …. I really like to receive information and like the percentage and the statistics of success rate I think is helpful for me personally.”

Subtheme: Incorporation of Medical and Pregnancy History Into Decision-Making

The participants had various medical and pregnancy complications that factored into their decision. For those who opted for CS, ECV seemed to pose unnecessary risk, given their medical history. One participant’s fetus was diagnosed in utero with Turner syndrome, and the participant explained that CS was the best option for them because their fetus would immediately be sent to the neonatal intensive care unit (NICU) postnatally for a cardiac procedure. Another participant described having undergone in vitro fertilization to achieve this pregnancy and wanted to minimize the number of additional interventions prior to delivery. For the other participants who opted for ECV, CS posed a greater risk to their health, given various medical conditions, such as a complex surgical history:

“I was like in a bad car accident like 10 years ago where I've had 15 surgeries from that alone and I have a history of blood clots, so obviously C-section puts you at a higher risk so like complications after it, especially with like blood clotting, which has been like a theme throughout my whole pregnancy. Yeah, managing that, and so I think like for me it's kind of like worth it to try the ECV. I think if I didn't have like all this medical history, like maybe I wouldn't go for the ECV.”

Theme 2: barriers to decision on malpresentation management

Subtheme: Lack of Information on ECV

The participants described having several unanswered questions about ECV after leaving their prenatal visits. Many vocalized their desire for additional information on potential complications to make more informed decisions. The overall uncertainty about ECV left the participants concerned about the complications of the procedure.

“They didn't really talk of all the complications to be honest, both of them.”

“I think just knowing all of it, to be honest, to give somebody their best chance at a decision for them and their family is really helpful. I think it's good to know all of the risks, as far in advance as possible to be honest not just day of.”

Among those who opted for the ECV, the participants desired more detailed information about the step-by-step process of the procedure. The participants acknowledged providers giving them opportunities to ask questions; however, the patients felt too uninformed to know what questions to ask. The patients favored standardized counseling that was patient-centric but provider-driven. For example, many participants wanted to know whether they could have partners or family in the procedure room or how long they would stay in the hospital after the ECV (Table 3). In addition, some participants stated they were not counseled on the ECV anesthesia options and how that would affect their hospital length of stay.

Theme 3: priorities and values for participants

Subtheme: Maternal Prioritization of Fetal Safety

Some patients approached the decision from the perspective of relative risk to their fetus. The participants vocalized that their uncertainty about ECV led them to believe it may be riskier than CS:

“I mean, the first provider sent me an article to read, which I did. But it still… I don't know, it still feels risky. Maybe it is not actually as risky, so if they had talked through the complications, I wouldn't see it as being as risky because the C-section is a major surgery too, so.”

“It just feels like the most respectful thing for myself and for my baby to just proceed with the C-section. I don't know I think it's just my comfort level with an ECV at present.”

Other participants vocalized the belief that perhaps there was a reason behind the fetus’ malposition, and an attempt to manipulate the fetus would pose unnecessary threat to fetal health. On the other hand, some participants felt that they were adequately counseled on the low likelihood of complications with ECV and expressed confidence that ECV would be a safe method to avoid CS (Table 3).

Subtheme: Desire to Avoid C-section

The desire to avoid CS was a prevalent theme. Several participants described CS as a major abdominal surgery and expressed concerns about balancing the recovery from surgery with caring for a newborn after CS. Furthermore, many participants who opted for ECV explained that they wanted to avoid CS so that they could experience a vaginal birth, which they viewed as a more “natural” option.

“I want don’t want to put more stress on my body than is necessary and I want to be present in the birth of my child - and to me, a C-section, if it’s necessary, it’s necessary. Unless medical need makes that my only option, I want to exhaust other options.”

Finally, for participants with other children delivered by vaginal birth, vaginal birth represented a familiar option that they had already experienced, compared to a CS (Table 3).

Theme 4: methods of malpresentation management beyond ECV or CS

Several participants expressed interest in alternative methods to convert their fetuses to vertex presentation. Approaches mentioned were moxibustion, acupuncture, chiropractor, use of a birthing ball, or strategies described by the Spinning Babies website (Table 3). Resources used were varied, so individual subthemes were not prominent; however, the broader theme arose as the participants frequently expressed trying various methods to avoid the need of a medical intervention for management of fetal malpresentation.

“And I also, in the course of preparing for the ECV, I was encouraged by one of the mid-wives to get acupuncture done. I also went to a chiropractor. She worked on the alignment of my hips in my pelvis … And that’s my understanding of it basically. He could flip on his own, but we are doing what we can to avoid the necessity of a C-section.”

## Discussion

This study examined the experiences of pregnant individuals as they navigated the process of deciding how to manage fetal malpresentation. Our findings arise in the background of international research on malpresentation management counseling [10-14] and reflect similar themes of patients’ preference for comprehensive safety data and detailed information about procedures and surgeries in order to make informed decisions. We found patients diagnosed with malpresentation desire expanded ECV counseling to facilitate their decision to pursue ECV versus CS. Specifically, patients desire more granular information regarding outcomes and risks, counseling focused on fetal safety, and details of the ECV procedure itself.

Thoroughly educating patients about options during their pregnancy protects patients’ agency over their health. The diagnosis of fetal malpresentation often arrives at a time of high stress, as individuals approach the end of their third trimester and are preparing for birth. Both CS and ECV can be frightening or undesirable for patients who have not been adequately informed about the risks and benefits. As one participant described, “I'd like to know what's happening to my body. So, the same thing goes with like learning about what's happening to my baby. I want to know as much information as I can.”

The patients underscored a preference for statistics during counseling to better understand the safety and success rates of ECV, given its unfamiliarity to many. Many participants found it easier to decide once they had learned of the ECV success rate and the relatively low risk of complications [15-17]. The participants mentioned providers reporting a 50% ECV success rate, despite meta-analyses demonstrating a success rate range of 16-100%, with a compiled success rate of 58% [15,17-19]. Neuraxial anesthesia has been shown to increase the ECV success rate and reduce maternal discomfort. However, some participants stated that they were not counseled on the ECV anesthesia options [20]. Ultimately, the goal for clinicians is not to bias individuals but provide patients with information and tools to make an informed decision.

Many participants cited concern for the safety of the fetus as a reason for opting for CS. Interestingly, some participants felt that there was a reason their baby was malpresenting and that it was meant to be. As a result, some participants felt that any effort to move the baby through ECV would put the baby’s life at risk. However, a prospective study measuring the outcomes of ECV on 805 women with fetal malpresentation found a 0.1% perinatal mortality rate with no fetal deaths associated with the procedure itself, indicating that ECV poses minimal risk to the fetus [11]. Providers should clarify misconceptions about the negative impact of ECV on the fetus.

Research is limited on how often providers in the US counsel on ECV and how prepared providers feel in doing so. Current limitations on providers to comprehensively counsel patients are important to acknowledge, as counseling may be restricted within the time constraints of a routine prenatal visit. Furthermore, the diagnosis of malpresentation often occurs within one to two weeks of optimal timing of ECV, typically 37 weeks, leaving a short window for subsequent counseling. Depending on the practice, different types of providers may be counseling patients, including some providers who may not perform ECVs themselves. Equipping clinicians with tools to improve counseling will empower patients to make informed decisions. To this end, clinics can prepare standardized materials, such as pamphlets or electronic medical record templates (e.g., smart phrases). This will ensure that all patients receive a thorough review of malpresentation and management options. Providers should be aware of alternative therapies for malpresentation, albeit with uncertain efficacy, as patients may ask questions about these options during counseling. In addition, there should be increased efforts to facilitate opportunities for patients to follow up with a provider in the short term, even if only by telephone, after the diagnosis of malpresentation and before the intended intervention.

The limitations of this study include the lack of racial and ethnic diversity among the participants, with 90% of the participants identifying as White and 100% of participants identifying as non-Hispanic. In addition, due to the lack of translation resources, this study excluded non-English-speaking individuals, despite Johns Hopkins Hospitals serving a large Spanish-speaking population. Individual experiences with healthcare and counseling are heavily influenced by background, such as race and language. Thus, the lack of diversity limits the generalizability of these findings as the study is focused on describing patient perspectives on counseling received. Additional research is warranted to describe the experience of non-White and non-English-speaking pregnant individuals diagnosed with fetal malpresentation.

## Conclusions

Overall, many pregnant individuals feel inadequately counseled and unprepared to make informed decisions regarding management of fetal malpresentation. Obstetric providers should aim to standardize their counseling on management of malpresentation and include quantitative data about the risks of ECV and CS. Counseling should be patient-centric, taking into consideration specific features of patients’ medical histories and birth preferences. Further research into specific systems-based practice interventions to achieve this for English and non-English-speaking patients are warranted. Optimizing ECV counseling will allow healthcare teams to transfer more agency back to pregnant individuals regarding management of malpresentation.
